# Experimental Investigation on the Flow Boiling of Two Microchannel Heat Sinks Connected in Parallel and Series for Cooling of Multiple Heat Sources

**DOI:** 10.3390/mi14081580

**Published:** 2023-08-10

**Authors:** Zhengyong Jiang, Mengjie Song, Jun Shen, Long Zhang, Xuan Zhang, Shenglun Lin

**Affiliations:** 1 Department of Energy and Power Engineering, School of Mechanical Engineering, Beijing Institute of Technology, Beijing 100081, China; 2 Department of Environmental Engineering, National Cheng Kung University, Tainan 70101, Taiwan; z11208011@ncku.edu.tw

**Keywords:** microchannel heat sinks, flow boiling, series connection, parallel connection, heat transfer coefficient, wall temperature, pressure drop

## Abstract

Cooling methods for multiple heat sources with high heat flux have rarely been reported, but such situations threaten the stable operation of electronic devices. Therefore, in this paper, the use of two microchannel heat sinks is proposed, with and without grooves, labeled Type A and Type B, respectively. Experimental investigations on the flow boiling of two microchannel heat sinks connected in parallel and in series are carried out under different mass fluxes. In addition, a high-speed camera is used to observe flow patterns in the microchannels. The cold plate wall temperature (*T*_w_), heat transfer coefficient (*HTC*), and pressure drop (*PD*) are obtained with the use of two microchannel heat sinks. The flow patterns of the bubbly flow and elongated bubbles in the microchannels are observed. The results of the analysis indicated that the *T*_w_, *HTC*, and *PD* of the two microchannel heat sinks connected in parallel were degraded, especially when using the Type A-B parallel connection. Compared to the use of a single heat sink, the maximum decrease in *HTC* was 9.44 kW/(m^2^K) for Type A heat sinks connected in parallel, which represents a decrease of 45.95%. The influence of the series connection on the *T*_w_, *HTC*, and *PD* of the two heat sinks is obvious. The Type A-A series connection exerted the greatest positive effect on the performance of the two heat sinks, especially in the case of the postposition heat sink. The maximum increase in *HTC* was 12.77 kW/(m^2^K) for the postposition Type A heat sink, representing an increase of 72.88%. These results could provide a reference for a two-phase flow-cooling complex for multiple heat sources with high heat flux.

## 1. Introduction

From the chip level to the system level, there are an enormous number of high-power electronic modules that are widely used to perform multi-threaded tasks requiring high strength, such as in supercomputing [1,2], high-power lasers, automotive power battery packs [3], phased-array radar, etc. [4]. To ensure stability and reliability, the problem of cooling multiple heat sources with high heat flux needs to be addressed. As stated, eliminating high heat flux from multiple heat sources is important, but research on relevant strategies have rarely been reported [5]. At present, traditional air and liquid-flow cooling strategies are widely used to cool electronics [6]. Air cooling is typically used when the heat flux is lower than 50 W/cm^2^. Liquid-flow cooling can be used to address heat flux reaching 100 W/cm^2^ but with a greater degree of uniformity of temperature between the inlet and outlet [7,8]. Furthermore, the heat flux of highly integrated electronic devices can reach 1000 W/cm^2^ [9]. The traditional cooling methods are not able to meet the heat dissipation of these high-heat-flux devices. Moreover, cooling problems for multiple heat sources with high heat flux are more challenging to overcome. Hence, there is a pressing need to develop and test advanced cooling techniques. One efficient method for solving high-heat-flux cooling problems is microchannel flow boiling [10,11].

Two-phase flow cooling has a higher heat transfer capacity and lower coolant flow velocity compared to single-phase flow [12,13]. This is because it mainly utilizes the latent heat of the coolant to absorb and carry away large amounts of heat. Due to the rapid evaporation of liquid, a large number of bubbles are produced, which are accompanied by variable and complex flow patterns [14,15]. In addition, the heat transfer properties of flow boiling in microchannels are strongly dependent on the flow pattern [16,17]; for example, the liquid–vapor flow distribution in the microchannels can affect the heat transfer capacity and pressure drop of flow boiling [18]. In addition, when flow boiling occurs in a long tube, there are several regions with increased superheat [10]. It has been reported that flow boiling processes are classically divided into five categories, including liquid convection, subcooled boiling, saturated boiling, transition boiling, and film boiling [19]. In addition, saturated boiling has the highest heat transfer capacity. However, as the superheating increases, there is a critical heat flux. When the heat flux exceeds the value of the critical heat flux, the heat transfer capacity of the flow boiling is suppressed. As a result, many methods have been tested with the aim of enhancing heat transfer and critical heat flux, such as modifying the geometrical structure of the microchannel.

Flow boiling in microchannel heat sinks has been widely studied, and the structure of the microchannels has been optimized to further optimize flow boiling [20,21]. Flow boiling in a copper foam fin microchannel heat sink was investigated experimentally by Fu et al. [20]. The results showed an 80% improvement in *HTC* and a 25% improvement in critical heat flux compared to the solid fin microchannel heat sink. Similarly, two microchannel heat sinks made of porous copper and solid copper were investigated by Yin et al. in flow boiling experiments [21], and the experimental results showed that the *HTC* of the porous open-microchannel was greatly improved compared with that for the solid copper open-microchannel heat sink. In addition, as reported by Li et al. [22], the critical heat flux was increased by 33.8~57.2% in a bidirectional counter-flow microchannel heat sink compared to the parallel-flow microchannel heat sink. A novel porous heat sink with reentrant microchannels was developed by Deng et al. [23], and the test results showed that the *HTC* value of the porous reentrant microchannels was 2–5 times higher than that of the solid copper microchannels. Furthermore, a bidirectional counter-flow microchannel heat sink was designed, and experimental study was performed [24,25]. The results showed that the average *HTC* of these novel microchannels was 33.5~62.0% higher than the traditional parallel-flow microchannels [24,25]. Subsequently, as reported by Jiang et al. [26], the counter-flow microchannels were modified with expanding angle, and flow boiling was performed in this heat sink at a higher heat flux of 2677 kW·m^−2^. Flow boiling has been extensively studied in microchannel heat sinks, and it is expected that it will be useful for cooling multiple heating sources with high heat fluxes.

Although flow boiling in microchannel heat sinks has been widely studied as a highly promising high-flux cooling method, only single-heat-source cooling has routinely been considered. Multiple-heat-source cooling problems are frequently encountered in advanced technology, and urgently need to be solved. Xu et al. [27] fabricated a cold plate with four microchannel regions made of copper to cool four heat sources. The experimental results showed that the temperature distribution of electronic devices with multiple heat sources was focused on single-phase flow. However, single-phase flow cooling was not a better method for high heat flux dissipation. Conversely, Tan et al. [28] modeled heat sources as point sources using the Dirac Delta function, and then extended this to locate the positions of the multiple heat sources in order to optimize the heat pipe performance. Similarly, an improved quasi-dynamic multiple-heat-source model was developed by Dan et al. [7] with the aim of optimizing the temperature distribution of a vapor chamber with multiple heat sources. It can be seen there are a small number of studies considering multiple heat sources with a vapor chamber in mathematical modeling. Recently, Zhang et al. [5] considered flow boiling in parallel/tandem microchannel heat sinks for cooling multiple heat sources. The experimental results showed that the phase transition heat of the upstream heat sink had a strong effect on the downstream heat sink. The connection of heat sinks is important for flow boiling with the aim of cooling multiple heat sources with high heat flux, but there is a lack of research results that would help to better understand this cooling method. As discussed, the studies on the cooling of multiple heat sources have rarely been reported, and more studies are needed to address the cooling problems related to multiple heat sources with high heat flux.

**Figure 1:**
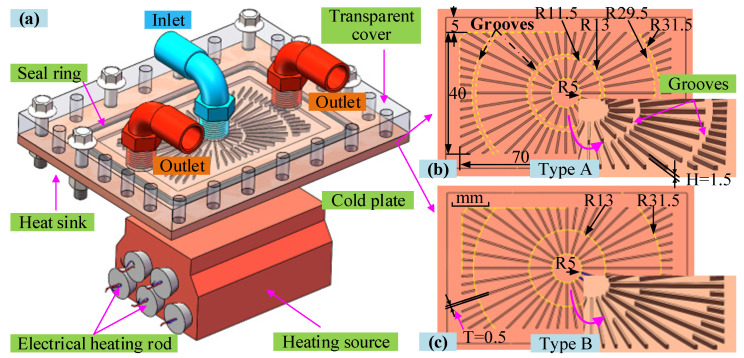
Diagram of the heat sink assembly with heating source and two cold plates: (**a**) the heat sink assembly with heating source; (**b**) microchannel cold plate with grooves; (**c**) microchannel cold plate without grooves.

The importance of removing high heat flux from multiple heat sources has been widely reported, but research into relevant strategies is rare. Therefore, in this study, a method is proposed based on two efficient rectangular radial expanding microchannel heat sinks with and without grooves, designed previously [29], which were labeled as Type A and Type B, respectively. Figure 1a presents the rectangular radial expanding microchannel heat sink assembled with a heat source. In addition, two cold plates with and without grooves are shown in Figure 1b,c. In the present work, two microchannel heat sinks are connected in either series or parallel, and share a circulating system in order to perform experimental studies on flow boiling at a fixed heating load of 300 W. The *T*_w_, *HTC*, and *PD* characteristics are obtained for the two heat sinks under different connection modes with different mass fluxes. The main corresponding flow patterns were observed using a high-speed camera. In addition, these characteristics are analyzed and compared in order to understand flow boiling in the radial expanding microchannels. Furthermore, the performances of the two heat sinks in different connection modes are compared in order to determine the most efficient connection mode for multiple-heat-source cooling. The results are expected to provide a reference for solving the high-heat-flux and complex multiple-heat-source cooling problems, thus improving the reliability of highly integrated electronic devices.

## 2. Materials and Methods

### 2.1. Experimental System and the Connection of Two Heat Sinks

**Figure 2:**
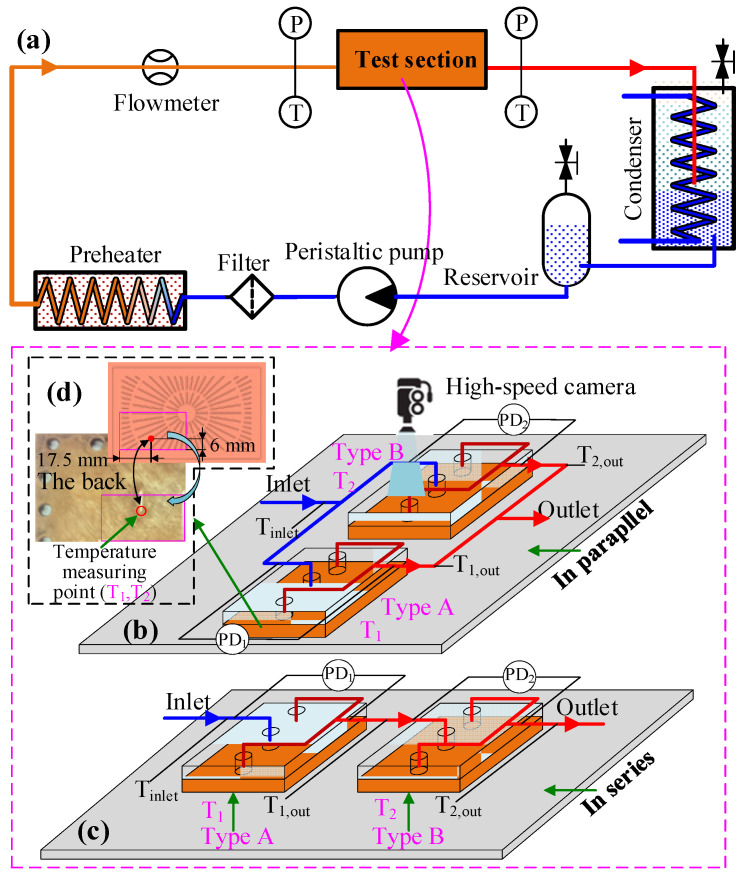
Diagrams of the flow boiling system and two heat sinks connected in parallel or series: (**a**) two-phase cooling system; (**b**) two cold plates connected in parallel; (**c**) two cold plates connected in series; (**d**) wall temperature measurement point.

The schematic diagram of a two-phase cooling system is presented in Figure 2a. The main experimental equipment includes a peristaltic pump, a flowmeter, a test section of the heat sink with the heat source, two thermostatic water baths, a reservoir, and a filter. In the test section, two heat sinks are connected in series or parallel to experimentally investigate flow boiling and to further analyze the performance of the two heat sinks. For the two heat sinks, one cold plate with annular grooves at the downstream microchannel was labeled Type A, while the other one without grooves was labeled Type B, as shown in Figure 1b,c, respectively. In addition, details regarding the size of the two microchannel cold plates can be found in refs [30,31]. The two heat sinks connected in parallel and series are shown in Figure 2b,c, respectively. Subsequently, the locations at which the temperature and *PD* parameters of the two heat sinks connected in parallel are measured are indicated in Figure 2b, as well as the inlet temperature of the working medium, *T*_inlet_, the outlet temperature of the working medium *T*_1,out_, *T*_2,out_, the wall temperature of the two cold plates, *T*_1_, *T*_2_, and the *PD* of the two heat sinks, *PD*_1_, *PD*_2_. Similarly, the locations at which the parameters for two heat sinks connected in series are measured are marked in Figure 2c. Real-time temperature and *PD* data are collected at 1 s intervals using an Agilent data acquisition instrument. In addition, the location of the cold plate wall temperature measurement point is shown in Figure 2d, and there is a groove with a depth of 1 mm in the back of the cold plate for welding the thermocouple measurement points. Furthermore, a high-speed camera is positioned directly above the heat sink in order to visually record the flow states, boiling bubbles, and flow patterns in the microchannels.

### 2.2. Experimental Procedures and Conditions

The two-phase cooling system is presented in Figure 2a. The two-phase flow medium from the heat sink flows directly into an open cooling box in a short pipeline to cool the liquid and condensate steam. In order to evaluate the *T*_w_, *HTC*, and *PD* of the two heat sinks in different connection modes at ambient atmospheric pressure, initially, the working medium consisted of deionized pure water. The heating load was set to a constant value of 300 W. The heating source was controlled separately using the voltage regulator and insulated with asbestos. The voltage ammeter was used to measure the power in real time. The water temperature at the inlet of the heat sink was kept constant at 88 °C (±0.2 °C) by a preheater, and a thermostatic water bath was used as the preheater. Subsequently, two thermocouples were used to monitor the inlet temperature at the inlet of the heat sink. In addition, 45 experimental cases were then designed under different mass fluxes with different connection modes.

As shown in Table 1, for two heat sinks connected in parallel, the main pipe volume flow rate was changed from 0.12 L/min to 0.40 L/min, with a gradient of 0.04 L/min, while the heating loads of the two heat sinks were kept constant at 300 W. In particular, the volume flow rates of two heat sinks connected in parallel were adjusted to be consistent before the phase change. Meanwhile, for the two heat sinks connected in series, the volume flow rate was changed from 0.12 L/min to 0.32 L/min and the heating loads of the two heat sinks was 300 W. In addition, the mass flux, *G*, was calculated using Equation (1) on the basis of the volume flow rate listed in Table 1. In addition, the *T*_w_, *HTC*, and *PD* characteristics for the two heat sinks were then experimentally investigated under different mass fluxes. The volume flow rate was measured before liquid boiling, and was controlled by changing the rotational speed of the peristaltic pump. Therefore, the heating loads were turned off to measure the next volume flow rate. Due to the delay in the increase in the cold plate wall temperature, it generally took about 4 min for the heat sink to reach the heat transfer balance. When the trend of *T*_w_ remained unchanged, or *T*_w_ was no longer increasing, the heat sink reached the heat transfer balance. The time of the heat transfer balance must last for at least 5 min. In addition, the data of the temperature and *PD* were then calculated by averaging the data over 4 min.

### 2.3. Calculated Parameters and Uncertainties

Firstly, the averaged mass flux, *G*, in the expanding microchannel was calculated using Equation (1).

(1) $$G=\frac{M}{2}\left(\frac{1}{A_{entr}}+\frac{1}{A_{\mathrm{exit}}}\right)$$

where *M* is the mass flow rate, *A*_in_ is the total cross-sectional area of the channel entrance, at 3.21 × 10^−5^ m^2^, and *A*_exit_ is the total cross-sectional area of the channel exit, at 2.88 × 10^−4^ m^2^.

Then, the heat flux, *q*_eff_, was calculated using Equation (2), and the *HTC* of the heat sink was calculated on the basis of the average cold plate wall temperature using Equation (3).

(2) $$q_{\mathrm{eff}}=\frac{Q}{A}$$

(3) $$HTC=\frac{q_{e}{}_{\mathrm{ff}}}{\left(T_{w}-T_{\mathrm{sat}}\right)}$$

where *Q* is the heating load, at 300 W, and *A* is the total efficient heat transfer area, which includes the base area and fin area. The areas of the Type A and Type B heat sinks are 4.868 × 10^−3^ m^2^ and 5.033 × 10^−3^ m^2^, respectively. *T*_w_ is the cold plate wall temperature measured by the thermocouple sensor, and *T*_sat_ is the saturated temperature in the heat sink, where the value of the *T*_sat_ is represented by the outlet temperature of the heat sinks.

The heat loss of the heat sink was determined, using the increase in the temperature for single-phase flow, as flow:

(4) $$\varphi_{loss}=\left[Q-C_{p}M\left(T_{\mathrm{out}}-T_{\mathrm{in}}\right)\right]/Q$$

where *C*_p_ is the specific heat capacity of water, at 4.2 × 10^3^ J/(kg·k), and *M* is the mass flow. *T*_out_ is the liquid temperature at the outlet of the heat sink, and *T*_in_ is the inlet temperature of the liquid.

All the measuring sensors were calibrated before testing. In addition, the dimensions of the microchannel are ±0.01 mm. The uncertainty of *HTC* was obtained using Equation (5).

(5) $$\frac{\partial HTC}{HTC}=\pm \sqrt{{\left(\frac{\partial U}{U}\right)}^{2}+{\left(\frac{\partial I}{I}\right)}^{2}+{\left(\frac{\partial A}{A}\right)}^{2}+{\left(\frac{\partial T_{wall}}{T_{wall}}\right)}^{2}+{\left(\frac{\partial T_{out}}{T_{out}}\right)}^{2}}$$

The maximum measured heat loss was 5.68%. The uncertainty of the calculated *HTC* was 7.68%. Similarly, the uncertainties of the other measured parameters were obtained using the same calculation method. The uncertainties for the other measurement parameters are listed in Table 2 with measuring ranges and errors.

### 2.4. Comparison with Existing Correlations

The experimental *HTC*s of the two Type A and Type B heat sinks, used individually, were compared to existing correlations under different mass fluxes. As listed in Table 3, the *HTC* correlations of Kandlikar [32], Fang and Zhou [33], and Fang and Wu [34] were used. Initially, these *HTC* correlations were applied to the straight microchannels, while the flow rate and the hydraulic diameter in the expanding microchannels were calculated on the basis of the average. In addition, the *HTC* in the two expanding microchannel cold plates was calculated in three stages when the fins changed. The experimental *HTC* was compared with the predicted *HTC* calculated using the three correlations. The calculation of the mean absolute deviation (*MAD*) is shown in Table 3. It can be seen that the *MAD* of the *HTC* between the experimental and the predicted results for a single Type A heat sink is within 30%, while the smallest *MAD* was 21.67%, and was obtained using the correlation of Fang and Zhou. For a single Type B heat sink, the *MAD* is within about 20%, and the best was 6.00%, using Kandlikar’s correlation. Therefore, based on existing *HTC* correlations, the experimental *HTC*s of the two heat sinks were first compared, in order to show a certain degree of accuracy under different mass fluxes at a heating load of 300 W.

## 3. Results

Based on the above experimental steps, *T*_w_, *PD*, *HTC*, and the corresponding flow patterns were obtained at different mass fluxes. Subsequently, these characteristic parameters of *T*_w_, *PD*, and *HTC* were analyzed. The influences of the parallel and series connections of the two heat sinks on the values of *T*_w_, *PD*, and *HTC* were quantified. In addition, the influence of the connection modes on Type A and Type B heat sinks was compared. 

### 3.1. Results for Two Heat Sinks Connected in Parallel

**Figure 3:**
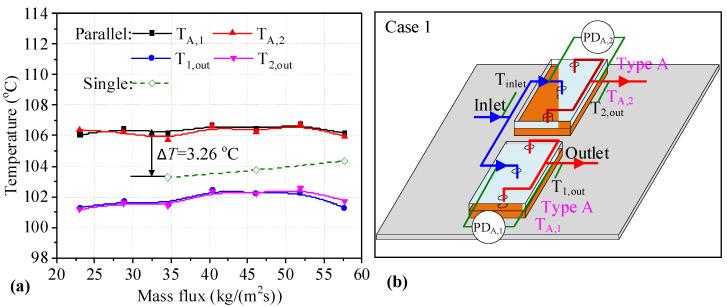
Temperatures of two heat sinks and measurement parameters in Case 1. (**a**) temperatures of two heat sinks; (**b**) the layout of the measurement parameters.

Two Type A heat sinks connected in parallel are presented in Figure 3b, with the measurement parameters marked out, where the subscript 1 and 2 differentiate the measurement data for the two heat sinks. In Figure 3a, the solid curves indicate the temperature data for two Type A heat sinks connected in parallel. In addition, the dotted line is the *T*_w_ of a single Type A heat sink. The abscissa of Figure 3a is the mass flux for a heat sink. In addition, the volume flow rate of each heat sink is adjusted to be the same before liquid boiling. Thus, the main pipe volume flow rate is evenly distributed. It can be seen that the *T*_w_ of the two heat sinks connected in parallel is similar. When the mass flux is increased from 23.08 kg/(m^2^s) to 57.67 kg/(m^2^s), the change in the *T*_w_ is small for two heat sinks. However, the *T*_w_ values for two heat sinks connected in parallel are higher than those for single heat sinks at the same mass flux. For example, the maximum temperature difference is 3.26 °C under a mass flux of 34.60 kg/(m^2^s). In addition, the outlet temperature for two heat sinks connected in parallel first increases and later decreases with increasing mass flux. As analyzed, the *T*_w_ values for heat sinks utilizing the Type A-A parallel connection are degraded compared to those of single heat sinks.

**Figure 4:**
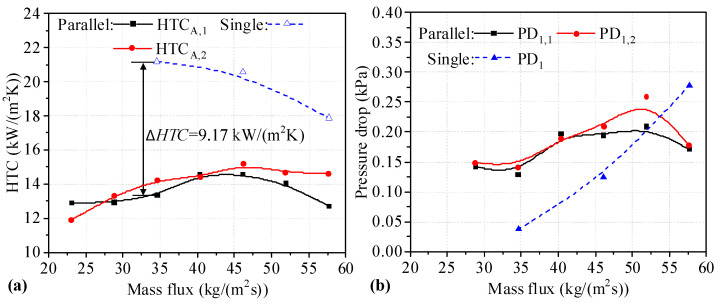
*HTC* and *PD* for two heat sinks utilizing the Type A-A parallel connection. (**a**) *HTC* of single and two parallel heat sinks; (**b**) *PD* of single and two parallel heat sinks.

The *HTC* values for two Type A heat sinks connected in parallel are shown in Figure 4a. It can be seen that the *HTC* for heat sinks connected in parallel first increases and later decreases. However, the *HTC* for single heat sinks decreases with increasing mass flux. The *HTC* values for heat sinks connected in parallel are lower than those for single heat sinks under the same mass flux. As shown, the maximum difference in *HTC* values between single heat sinks and those connected in parallel is 9.17 kW/(m^2^K). The decrease in the *HTC* is 43.32%. As shown in Figure 4b, the difference in *PD* between two Type A heat sinks connected in parallel is small, and the maximum difference occurs at a mass flux of 51.90 kg/(m^2^s). It can be seen that the trend of *PD* values of two heat sinks connected in parallel increases slightly until the mass flux reaches 51.90 kg/(m^2^s), and decreases sharply at the end. This may be because boiling is inhibited by higher mass flux. The *PD* values for heat sinks connected in parallel are higher than those for single heat sinks at mass flux values of 34.60 and 46.14 kg/(m^2^s). It can be seen that the *HTC* and *PD* values for two Type A heat sinks connected in parallel are degraded.

**Figure 5:**
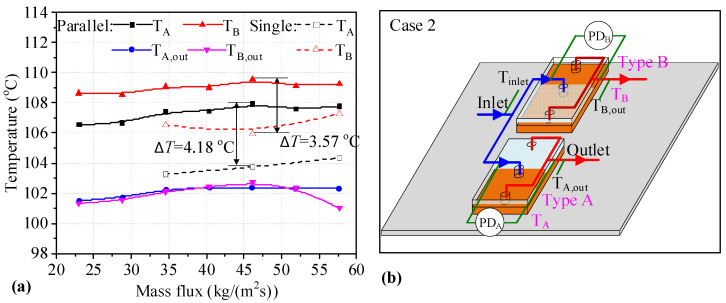
Temperatures of two heat sinks with the measured parameters marked in Case 2. (**a**) temperatures of two heat sinks; (**b**) the layout of the measurement parameters.

**Figure 6:**
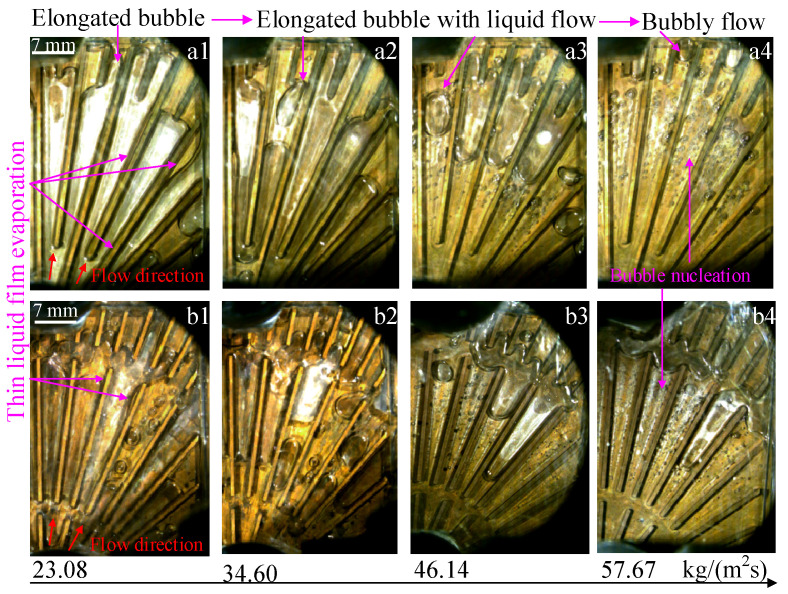
Flow patterns in two heat sinks under different mass fluxes in Case 2. (**a1**) flow patterns in channels without grooves at 23.08 kg/(m^2^s); (**a2**) flow patterns in channels without grooves at 34.60 kg/(m^2^s); (**a3**) flow patterns in channels without grooves at 46.14 kg/(m^2^s); (**a4**) flow patterns in channels without grooves at 57.67 kg/(m^2^s); (**b1**) flow patterns in channels with grooves at 23.08 kg/(m^2^s); (**b2**) flow patterns in channels with grooves at 34.60 kg/(m^2^s); (**b3**) flow patterns in channels with grooves at 46.14 kg/(m^2^s); (**b4**) flow patterns in channels with grooves at 57.67 kg/(m^2^s).

Figure 5b depicts two heat sinks of Types A and B connected in parallel, along with the measurement parameters. The temperatures measured for two heat sinks connected in parallel are shown in Figure 5a. It can be seen that the *T*_w_ values for two heat sinks of Types A and B increase slightly with increasing mass flux. The values are higher than those of single heat sinks, with the maximum difference between single Type A heat sinks and those connected in parallel being 4.18 °C, while for the Type B heat sink the maximum difference is 3.57 °C. The *T*_w_ values for the Type A heat sink are lower than those of the Type B heat sink when connected in parallel. The outlet temperatures of the two heat sinks are similar until the mass flux reaches 51.90 kg/(m^2^s). Meanwhile, the outlet temperature of the Type B heat sink decreases obviously. This may be because boiling is inhibited by higher mass flux, and these changes can be further explained by the flow patterns in Figure 6. It can be seen that the *T*_w_ values for two heat sinks of Types A and B connected in parallel are degraded.

The change in the flow patterns of two heat sinks connected in parallel is shown in Figure 6. In addition, the change in the flow patterns could reflect differences in the heat transfer mechanism under different mass fluxes. It can be seen that, when the mass flux increases from 23.08 kg/(m^2^s) to 57.67 kg/(m^2^s), the main flow patterns of two heat sinks connected in parallel correspond to elongated bubble flow, elongated bubble with liquid flow, and bubbly flow. In addition, this change in flow pattern indicates that the heat transfer mechanism has also changed, with thin liquid film evaporation occurring at a lower mass flux and bubble nucleation at a higher mass flux. Furthermore, the change in the heat transfer mechanism leads to a change in the *T*_w_ and *HTC* in a non-monotonic relationship with mass flux. At lower mass flux, the heat transfer mechanism is the evaporation of thin liquid film, which has a higher heat transfer capacity compared to the bubble nucleation heat transfer mechanism [35]. Thus, the *T*_w_ increases with increasing mass flux when the flow rates are low. Furthermore, the evaporation decreases at high mass flux, which may lead to a decrease in the outlet temperature of the medium.

**Figure 7:**
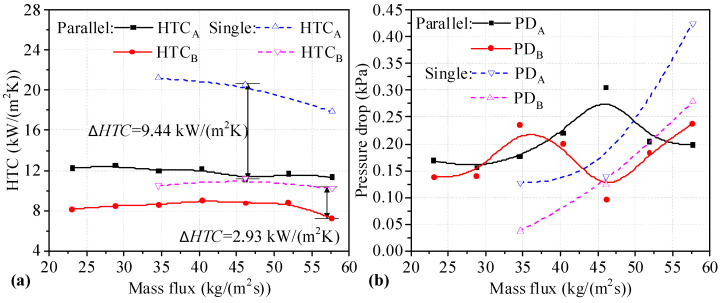
*HTC* and *PD* values for two heat sinks utilizing the Type A-B parallel connection. (**a**) *HTC* of single and two parallel heat sinks; (**b**) *PD* of single and two parallel heat sinks.

The *HTC* values of two heat sinks of Types A and B connected in parallel are shown in Figure 7a. It can be seen that the *HTC* values of the Type B heat sinks connected in parallel first increase and later decrease with increasing mass flux. The *HTC* values for Type A heat sinks connected in parallel decrease slightly with increasing mass flux. The *HTC* values for two heat sinks connected in parallel are lower than those of single heat sinks under the same mass flux. In addition, the difference in the *HTC* values between single Type A heat sinks and those connected in parallel is more obvious. It can be seen that the maximum difference in *HTC* values between single Type A heat sinks and those connected in parallel is 9.44 kW/(m^2^K) at a mass flux of 46.14 kg/(m^2^s). The decrease in *HTC* is 45.95%. For Type B heat sinks, the maximum difference in *HTC* values between single heat sinks and those connected in parallel is 2.93 kW/(m^2^K) at a mass flux of 57.67 kg/(m^2^s). As shown in Figure 7b, the *PD* values for two heat sinks connected in parallel are fluctuation, with one increasing and the other decreasing under the same mass flux. The *PD* change trend for two heat sinks connected in parallel shows a slight upward trend compared to single heat sinks. The *PD* for single heat sinks is lower than that of heat sinks connected in parallel at 34.60 and 51.90 kg/(m^2^s). It can be seen that the *HTC* values for two heat sinks connected in parallel is degraded, along with *PD*, at low mass flux.

**Figure 8:**
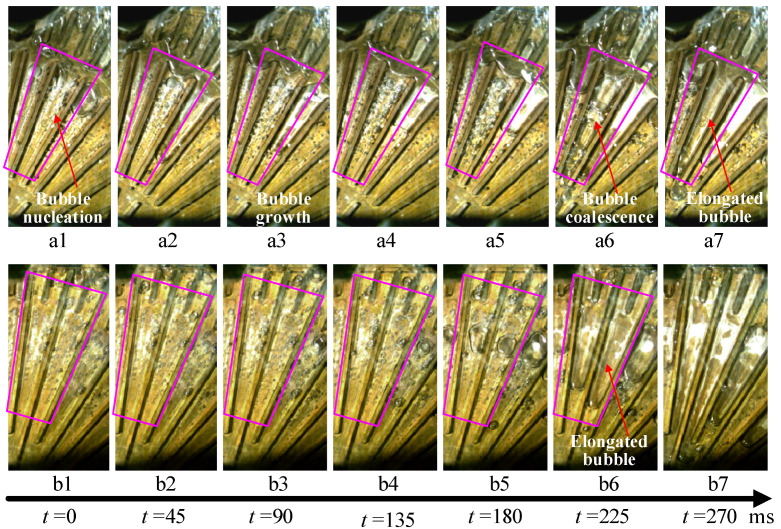
Main flow patterns in microchannels of the two heat sinks in Case 2. (**a1**) flow patterns in channels with grooves at t = 0 ms; (**a2**) flow patterns in channels with grooves at t = 45 ms; (**a3**) flow patterns in channels with grooves at t = 90 ms; (**a4**) flow patterns in channels with grooves at t = 135 ms; (**a5**) flow patterns in channels with grooves at t = 180 ms; (**a6**) flow patterns in channels with grooves at t = 225 ms; (**a7**) flow patterns in channels with grooves at t = 270 ms; (**b1**) flow patterns in channels without grooves at t = 0 ms; (**b2**) flow patterns in channels without grooves at t = 45 ms; (**b3**) flow patterns in channels without grooves at t = 90 ms; (**b4**) flow patterns in channels without grooves at t = 135 ms; (**b5**) flow patterns in channels without grooves at t = 180 ms; (**b6**) flow patterns in channels without grooves at t = 225 ms; (**b7**) flow patterns in channels without grooves at t = 270 ms.

Boiling bubbles developed for the two heat sinks when utilizing a Type A-B parallel connection, as shown in Figure 8. The boiling bubbles grow and exit periodically in the downstream microchannels of two heat sinks. As shown in the area marked in the red box, it takes approximately 270 ms for the bubbles to nucleate and grow to completely fill in the microchannels at a heating load of 300 W and a mass flux of 46.14 kg/(m^2^s). For the Type A heat sinks connected in parallel, the bubble first grows in the downstream annular grooves and then extends into adjacent channels. Subsequently, the bubbles coalesce in the middle channel to form a large bubble that fully fills the microchannel. For the Type B heat sink, the bubbles nucleate in downstream microchannels, and then the bubbles coalesce first in the middle microchannels. Subsequently, the coalescent bubbles first extend downstream, and upstream later. The development of the boiling bubbles in microchannels reflects the heat transfer state of two heat sinks. The main flow patterns are the bubbly flow and the elongated bubbles in the microchannel. The main heat transfer mechanism is bubble nucleation, and liquid boiling evaporation absorbs heat for bubble growth.

**Figure 9:**
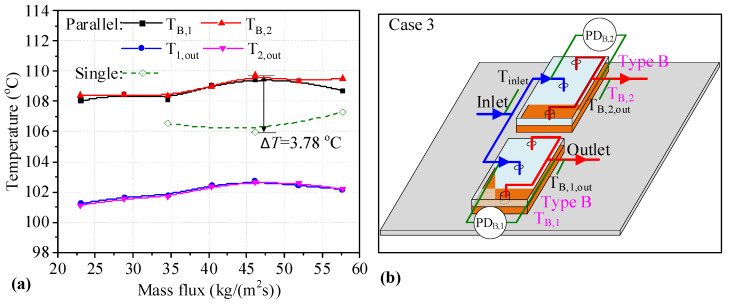
Main flow patterns in microchannels of two heat sinks in Case 2. (**a**) temperatures of two heat sinks; (**b**) the layout of the measurement parameters.

Figure 9b presents a schematic diagram of two Type B heat sinks connected in parallel on which the measured parameters are marked. The temperatures for two heat sinks connected in parallel are shown in Figure 9a. It can be seen that *T*_w_ values of the two Type B heat sinks are similar, and they have a slightly increasing trend with the increase in mass flux. The values are higher than those for single heat sinks, with the maximum difference between the single Type B heat sinks and those connected in parallel being 3.78 °C. The outlet temperatures of two heat sinks first increase and later decrease. This may be because boiling is inhibited by a higher mass flux. It can be seen that the *T*_w_ for two Type B heat sinks connected in parallel is degraded.

**Figure 10:**
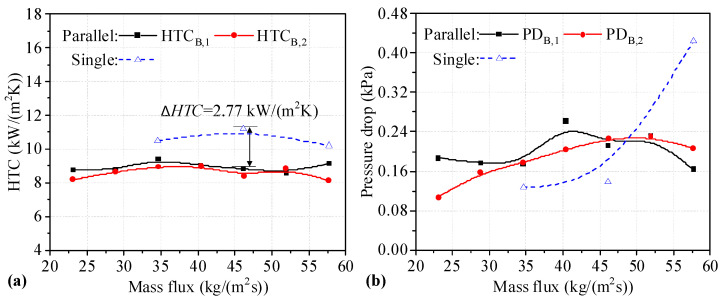
*HTC* and *PD* of two heat sinks in a Type B-B parallel connection. (**a**) *HTC* of single and two parallel heat sinks; (**b**) *PD* of single and two parallel heat sinks.

The *HTC* values of two Type B heat sinks connected in parallel are shown in Figure 10a. It can be seen that the *HTC* of heat sinks connected in parallel first increases and then decreases. The *HTC* values of the parallel heat sink are lower than those of single heat sinks under the same mass flux. It can be seen that the maximum *HTC* difference between the parallel and single heat sinks is 2.77 kW/(m^2^K) at a mass flux of 40.37 kg/(m^2^s). The decrease in the *HTC* is 24.72%. As shown in Figure 10b, the changing trend of *PD*_B,2_ in the heat sinks connected in parallel shows an upward trend, reaching a peak at a mass flux of 46.14 kg/(m^2^s), after which *PD*_B,2_ decreases slightly. Although the change trend of *PD*_B,1_ is fluctuant, the *PD* values in two Type B heat sinks connected in parallel are small, and the maximum value is about 0.27 kPa for *PD*_B,1_. This may be because boiling is inhibited by the higher mass flux. The *PD* of heat sinks connected in parallel is higher than that of single heat sinks at mass flux values of 34.60 and 46.14 kg/(m^2^s), and the difference in *PD* between single Type B heat sinks and those connected in parallel is small. However, the *PD* of single Type B heat sinks is significantly higher than that of heat sinks connected in parallel. It can be seen that the *HTC* values for two Type B heat sinks connected in parallel are degraded, but the *PD* values of two Type B heat sinks connected in parallel decrease with high mass flux.

Table 4 lists the mean values of *T*_w_, *HTC*, and *PD* for two heat sinks connected in parallel at different flow rates. It can be seen from the mean *T*_w_, *HTC*, and *PD* values for the two heat sinks that the performance of heat sinks is better when utilizing the Type A-A parallel connection compared to when utilizing the Type A-B and Type B-B parallel connections. In addition, the performance of the heat sink in the Type A-B parallel connection is the worst, where the *T*_w_ of the Type B heat sink is the highest and the *HTC* is the lowest. Therefore, when two heat sinks are connected in parallel, the two heat sinks should be of the same type in order to achieve a stable performance. In addition, as described above, the *T*_w_, *PD*, and *HTC* values of heat sinks connected in parallel are different from those of individual heat sinks, because there are mutual influences affecting the two heat sinks connected in parallel. This may be related to the fluctuation of the mass flux when phase change occurs in two heat sinks connected in parallel.

### 3.2. Results of Two Heat Sinks Connected in Series

**Figure 11:**
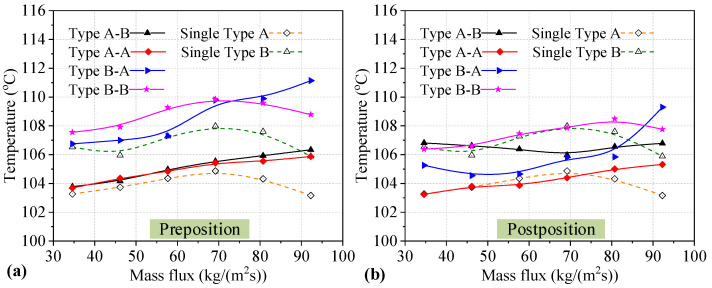
*T*_w_ of two heat sinks utilizing different series connections. (**a**) *T*_w_ of preposition heat sinks; (**b**) *T*_w_ of postposition heat sinks.

There are four series connection modes for two Type A or B heat sinks connected in series, as listed in Table 1. The *T*_w_ values determined for the preposition and postposition heat sinks after testing and analysis are shown in Figure 11a,b, respectively. The solid curves represent the data correspond to heat sinks connected in series, and the dotted lines belong to single heat sinks. As seen in Figure 11a, for the preposition heat sinks connected in series, the *T*_w_ in Type A heat sinks connected in series always shows an upward trend with increasing mass flux, while the *T*_w_ in Type B heat sinks connected in series is fluctuant and even exhibits different change trends with different series connections. In addition, the *T*_w_ of single heat sinks first increases, until the mass flux reaches 69.20 kg/(m^2^s), and then sharply decreases. In contrast, all the *T*_w_ values of preposition heat sinks utilizing different series connections are higher than those of single heat sinks. This may be because the flow boiling state in the two cold plates is affected by the series connection, increasing, for example, the pressure in the preposition heat sinks. Furthermore, all of the *T*_w_ values of Type A heat sinks are lower than those of the Type B heat sinks connected either in series or individually. The reason for this is that the grooves in the channels are beneficial for the outflow of large bubbles from the channel. The analysis showed that the difference in the value of *T*_w_ between the single Type A heat sinks and those connected in series is lower than that of Type B heat sinks. For preposition heat sinks connected in series, a lower *T*_w_ value was found for the Type A-A series connection compared to the other series connection modes. However, the lowest value of *T*_w_ observed for preposition heat sinks connected series is higher than that of single heat sinks. The *T*_w_ of two preposition heat sinks connected in series is degraded.

As shown in Figure 11b, for postposition Type A heat sinks connected in series, the *T*_w_ exhibits an upward trend with increasing mass flux in the Type A-A connection mode, while the *T*_w_ first decreases slightly, until a mass flux of 46.14 kg/(m^2^s) is reached, and then increases sharply for the Type B-A connection mode. For postposition Type B heat sinks connected in series, the *T*_w_ gradually increases until the mass flux reaches 69.20 kg/(m^2^s), and then it decreases slowly until the end in the Type B-B connection mode, while the *T*_w_ first decreases slightly until the mass flux reaches 69.20 kg/(m^2^s), before increasing slightly until the end with the Type A-B connection mode. In contrast, the majority of the *T*_w_ values of Type A heat sinks connected in series are higher than those of single heat sinks. Only at mass flux values of 57.67 kg/(m^2^s) and 69.20 kg/(m^2^s) are the *T*_w_ values of heat sinks in the Type A-A connection mode lower than those of single heat sinks. The majority of *T*_w_ values of postposition Type B heat sinks are higher than those of Type A heat sinks. The majority of *T*_w_ values of postposition Type B heat sinks in series are similar to those of single heat sinks, or even lower than those of single heat sinks. For postposition heat sinks connected in series, a lower *T*_w_ value is obtained for the Type A-A series connection compared to the other series connection modes. In addition, there are some *T*_w_ values for preposition heat sinks connected in series that are lower than those of single heat sinks. It can be seen that it was possible to optimize the *T*_w_ of preposition heat sinks connected in series.

**Figure 12:**
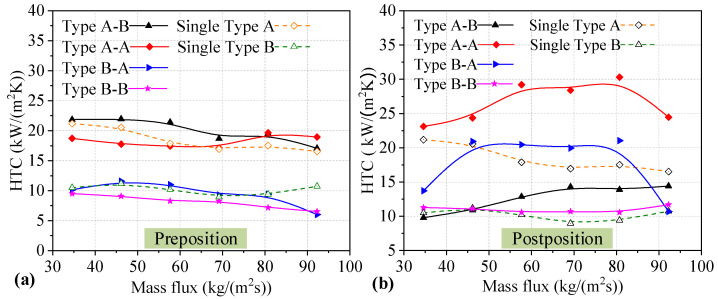
*HTC*s of two heat sinks utilizing different series connections. (**a**) *HTC* of preposition heat sinks; (**b**) *HTC* of postposition heat sinks.

Furthermore, for the *HTC* of two heat sinks utilizing the four series connection modes, the *HTC* values of preposition and postposition heat sinks are shown in Figure 12a,b, respectively. As shown in Figure 12a, for preposition Type A heat sinks connected in series, the *HTC* of the heat sink in the Type A-B connection mode shows a downward trend with increasing mass flux, while the *HTC* of the heat sink in the Type A-A connection mode decreases slowly first, and then increases slightly towards the end. For preposition Type B heat sinks connected in series, the *HTC* values of the heat sink in Type B-A and Type B-B connection modes show a downward trend with increasing mass flux. In contrast, the majority of *HTC* values in preposition Type B heat sinks connected in series are lower than those of single heat sinks. However, the majority of *HTC* values of Type A heat sinks are higher than those of single heat sinks. In addition, all the *HTC* values of Type A heat sinks are higher than those of Type B heat sinks with different mass fluxes. The grooves in the channel cause the differences in the performance of the two heat sinks. It can be seen that the difference in *HTC* between the single heat sinks and those connected in series is small. For preposition heat sinks connected in series, higher *HTC* values were found for the Type A-B series connection compared to the other series connection modes. In addition, the highest *HTC* value observed for preposition heat sinks connected in series is higher than that for single heat sinks. The effect of being connected in series on the *HTC* of two preposition heat sinks is small, and the *HTC* value of the Type A heat sink is optimal in the Type A-B series connection mode.

As shown in Figure 12b, for postposition Type A heat sinks connected in series, the *HTC* of heat sinks in the Type A-A and Type B-A connection modes increases at the beginning and maintains a constant at moderate mass flux, but decreases sharply at the end. For postposition Type B heat sinks connected in series, the *HTC* in the Type A-B connection mode increases slightly until the mass flux reaches 69.20 kg/(m^2^s), after which the *HTC* maintains a constant level. In addition, the *HTC* value of heat sinks in the Type B-B connection mode remains almost stable with increasing mass flux. In contrast, the majority of the *HTC* values of postposition heat sinks connected in series are higher than those of single heat sinks. In addition, the majority of *HTC* values of Type A heat sinks are higher than those of Type B heat sinks under different mass fluxes. It can be seen that a higher *HTC* value was obtained for the Type A-A series connection compared to the other series connection modes. In addition, the highest *HTC* value observed for preposition heat sinks connected in series is significantly higher than those of single heat sinks. The maximum increase in *HTC* is 12.77 kW/(m^2^K), at a mass flux of 80.74 kg/(m^2^s), which represents an improvement of 72.88% compared to a single Type A heat sink. The reason for the increase in *HTC* is that the heat transfer mechanism of the postposition heat sinks is mainly the thin liquid film evaporation mechanism. This mechanism is caused by the preposition heat sinks supplying a portion of the steam. The effect of being connected in series on the *HTC* of the two postposition heat sinks is obvious, and the *HTC* in two postposition heat sinks is optimal.

**Figure 13:**
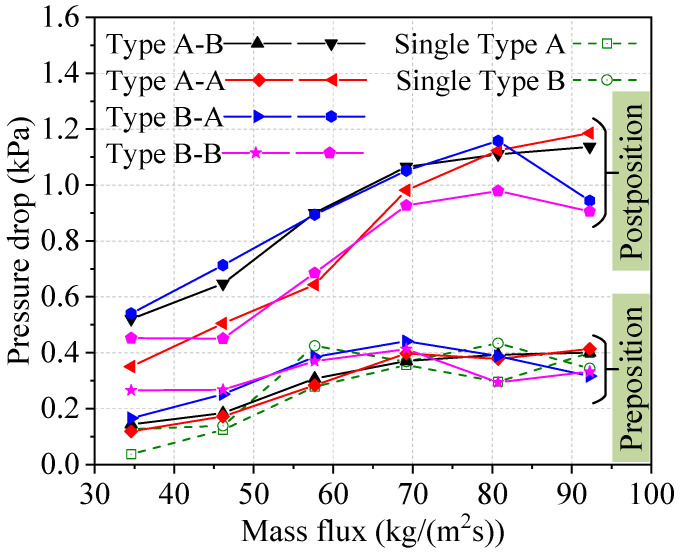
*PD*s of two heat sinks utilizing different series connections.

The change trends of *PD* in two heat sinks connected in series are shown in Figure 13. Firstly, the *PD* values for individual Type A and Type B heat sinks are similar, and have an upward trend. The *PD* of two single heat sinks increases when the mass flux changes from 34.60 kg/(m^2^s) to 57.67 kg/(m^2^s). When the mass flux exceeds 57.67 kg/(m^2^s), the *PD* of the two single heat sinks barely changes. These trends are similar to the change trend of the *PD* for preposition Type A and B heat sinks connected in series. Conversely, for postposition heat sinks connected in series, the *PD* increased significantly compared to single heat sinks. The analysis showed that the maximum difference in *PD* between the single heat sinks and the Type B heat sinks connected in series is 0.79 kPa, which represents an increase by about 200%. This is mainly because the flow boiling state in the postposition heat sink is more dramatic and postposition heat sinks have a higher *HTC* compared to preposition heat sinks. Therefore, the series connection has a significant impact on *PD* values in postposition heat sinks. In addition, the *PD* values of two heat sinks in the Type A-A and Type B-B series connections have lower values compared to other series connection modes. The *PD* for postposition heat sinks connected in series is increased significantly compared to single heat sinks, but the *HTC* for postposition heat sinks connected in series is higher.

### 3.3. Comparison of Two Heat Sinks Connected in Series and Parallel 

The mean differences in the *T*_w_, *HTC*, and *PD* between single heat sinks and two heat sinks connected in series or parallel at mass fluxes of 34.60, 46.14, and 57.67 kg/(m^2^s) are presented in Table 5. Firstly, the mean difference in the *T*_w_ for two heat sinks connected in parallel is about +2.18~+3.91 °C. This shows that the *T*_w_ of two heat sinks when using different parallel connections is obviously degraded. In addition, the *T*_w_ in the Type A-B parallel connection is the worst. The mean difference in the *HTC* for two heat sinks connected in parallel is about −1.50~−8.40 kW/(m^2^K). This shows that the *HTC* of two heat sinks utilizing different parallel connections is degraded; in particular, this is most obvious for the Type A heat sink. Meanwhile, the mean difference in the *PD* for two heat sinks connected in parallel is small. This shows that the effect of parallel connection on the *PD* for heat sinks is small.

Subsequently, the mean differences in *T*_w_ between preposition heat sinks in different series connections increase, which shows that the series connection degrades the *T*_w_ of preposition heat sinks connected in series. However, the effect on the *T*_w_ is small compared to when connected in parallel. For the postposition heat sinks connected in series, the *T*_w_ is optimal in the Type A-A series connection. In addition, the mean *HTC* differences between the preposition and postposition heat sink exhibit a small increase in the Type A-B series connection. The *HTC* in the Type A-A series connection shows the most obvious improvement. This shows that the *HTC* of the postposition heat sink can be optimized when connected in series, and that the effect of the series connection on the *HTC* of the preposition heat sink is small. The mean difference in the *PD* of the preposition heat sinks increases slightly when connected in series, but the increase is obvious for the postposition heat sinks. In addition, the difference in *PD* between the preposition and postposition heat sinks is up to 5~6 times larger. The series connection degrades the *PD* of two heat sinks utilizing different connection modes, especially in the case of postposition heat sinks. On the basis of an analysis of the mean difference, the Type A-A series connection has the greatest positive effect on the performance of two heat sinks connected in series, especially in the case of postposition heat sinks at moderate mass flux. However, the *T*_w_, *HTC*, and *PD* characteristics of the two heat sinks are been degraded when connected in parallel. Thus, the Type A-A series connection of two heat sinks is recommended for cooling multiple heat sources. In addition, when two heat sinks are connected in parallel, the two heat sinks should be of the same type in order to achieve stable performance.

## 4. Conclusions

Experimental investigation of flow boiling with two microchannel heat sinks connected in series and parallel for the cooling of multiple heat sources was performed. The following four conclusions were drawn in this study:

(1) An experimental investigation on flow boiling of two microchannel heat sinks connected in series and parallel was conducted, and the *T*_w_, *HTC*, and *PD* characteristics of two microchannel heat sinks utilizing different connection modes were obtained. The bubbly flow and elongated bubble flow patterns were observed. In addition, the effect on these characteristics of being connected in series versus in parallel was then analyzed.
(2) The parallel connection significantly affected the *T*_w_, *HTC*, and *PD* of the two heat sinks. The majority of these characteristics were degraded when connected in parallel. For example, compared to single heat sinks, the maximum increase in *T*_w_ was 4.18 °C for the Type A heat sinks connected in parallel, and the maximum decrease in *HTC* was 45.95%. The *PD* of two heat sinks connected in parallel was higher than that of a single heat sink.
(3) The influence of being connected in series on the *T*_w_ for two Type A and Type B heat sinks is obvious. The *T*_w_ in preposition heat sinks connected in series was degraded, but some of the *T*_w_ in postposition heat sinks connected in series could be optimized. The *PD* of the postposition heat sinks connected in series was significantly increased, but the *HTC* was optimized. For example, the maximum increase in *HTC* was 72.88% for the postposition heat sinks connected in series.
(4) The performance of two heat sinks in different parallel connections was degraded compared to that of a single heat sink. Meanwhile, the Type A-A series connection had the most positive effect in terms of the performance of the two heat sinks, especially in the case of the postposition heat sink with moderate mass flux. Thus, the Type A-A series connection is recommended for cooling multiple heat sources.

## Figures and Tables

**Table 1:** Forty-five experimental cases were designed at a fixed heating load of 300 W.

Types	No.	Heat Sink Type	Volume Flow Rates and Mass Fluxes
In parallel	Case 1	Type A–A	Main pipe volume flow rates (L/min): 0.16, 0.20, 0.24, 0.28, 0.32, 0.40 Corresponding mass fluxes (kg/(m^2^s)): 46.14, 57.67, 69.20, 80.74, 92.27, 103.81, 115.34
	Case 2	Type A–B	
	Case 3	Type B–B	
In series	Case 4	Type A–B	Volume flow rates (L/min): 0.12, 0.16, 0.20, 0.24, 0.28, 0.32 Corresponding mass fluxes (kg/(m^2^s)): 34.60, 46.14, 57.67, 69.20, 80.74, 92.27
	Case 5	Type A–A	
	Case 6	Type B–A	
	Case 7	Type B–B	

**Table 2:** The uncertainties of the measured and calculated parameters.

No.	Measured Parameters	Uncertainties
1	Volume flow rate (*q*_v_)	±0.69%
2	Heating load (*Q*)	±0.2%
3	Temperature (*T*_in_, *T*_out_, *T*_w_)	±0.2 K
4	Pressure drop (*PD*)	±0.25%
5	Heat transfer coefficient (*HTC*)	±7.68%
6	Mass flow (*M*)	±0.96%
7	Dimension (*A*, *H*, *D*)	±0.01 mm

**Table 3:** Mean absolute deviations between the experimental and predicted *HTC.*

No.	Correlations	^a^ Mean Absolute Deviations	
		Type A	Type B
1	Kandlikar	24.32%	6.00%
2	Fang and Zhou	21.67%	20.05%
3	Fang and Wu	26.40%	19.28%

^a^ Mean absolute deviation $=\frac{1}{N}\sum_{1}^{N}\left.\left|\frac{HTC_{\exp}-HTC_{\mathrm{pre}}}{HTC_{\exp}}\right.\right|$, where *N* is the number of data.

**Table 4:** Mean *T*_w_, *HTC*, and *PD* of two heat sinks connected in parallels.

No.	Parallel Connections	Right Heat Sink			Left Heat Sink		
		*T*_w_	*HTC*	*PD*	*T*_w_	*HTC*	*PD*
Case 1	Type A–Type A	106.38	13.59	0.18	106.17	14.48	0.16
Case 2	Type A–Type B	107.33	11.86	0.20	109.02	8.45	0.18
Case 3	Type B–Type B	108.75	8.94	0.20	108.97	8.60	0.19

**Table 5:** Mean differences in *T*_w_, *HTC*, and *PD* under mass flux of 34.60, 46.14, and 57.67 kg/(m^2^s).

**Parallel Connections**								
**No.**	**Left Heat Sink**			**Heat Sink Types**		**Right Heat Sink**		
	***T*_w_**	***HTC***	***PD***	**Left**	**Right**	***T*_w_**	***HTC***	***PD***
Case 1	+2.43	−5.81	+0.02	Type A–Type A		+2.46	−6.32	+0.03
Case 2	+2.69	−2.43	−0.04	Type A–Type B		+3.91	−8.40	+0.08
Case 3	+2.61	−2.12	−0.03	Type B–Type B		+2.18	−1.50	−0.05
**Series Connections**								
**No.**	**Preposition Heat Sink**			**Heat Sink Types**		**Postposition Heat Sink**		
	***T*** **_w_**	***HTC***	***PD***	**Pre.**	**Post.**	***T*** **_w_**	***HTC***	***PD***
Case 4	+0.52	+1.88	+0.06	Type A–Type B		+0.02	+0.56	+0.46
Case 5	+0.51	−1.90	+0.04	Type A–Type A		−0.15	+5.68	+0.35
Case 6	+0.43	+0.29	+0.04	Type B–Type A		+1.03	−1.50	+0.57
Case 7	+1.66	−1.67	+0.07	Type B–Type B		+0.15	+0.36	+0.30

The mean difference in *Tw* $=\frac{1}{N}\sum_{1}^{N}\left(T_{series}-T_{single}\right)$°C, and the mean differences in *HTC* kW/(m^2^K) and *PD* kPa are calculated using the same method.

## Data Availability

The research data for this article can be obtained from the corresponding author “Mengjie Song” on reasonable request.
